# Military Categories in Diseases of the Ear

**Published:** 1919

**Authors:** 


***Military Categories in Diseases of the Ear.*** By Captain JOHN F. O'MALLEY. *Journal of the Royal Army Medical Corps,* August, 1918.

When questions have to be repeated to a man while he is being examined, or the speaker's voice has to be raised, or a history of deafness with or without suppuration is given or ascertained, the case should pass on to the aural surgeon for examination.

Such cases can be grouped as follows :

1. Middle-ear deafness : (*a*) Middle-ear catarrh; (*b*) Otosclerosis; (*c*) Past suppurations, but membranes well healed and no recurrences for many years.

2. Internal ear deafness, which includes a family type.

3. Mixed middle and internal ear deafness : (*a*) Advanced middle ear catarrhal cases; (*b*) Artillery men who have been exposed to long periods of intense gun fire; (*c*) Some shell concussion cases, middle and internal ear lesions combined.

Grades of suppuration.

1. Some membrane loss, large or small, but the drainage is good. The middle ear cavity is dry, or only slightly moist with mucus or pus.

2. The membrane loss may be large or small, but the drainage is bad : (*a*) The membrane may show a small perforation, situated a considerable distance above the floor of the middle ear cavity. (*b*) The middle ear cavity is anatomically shallow and not a clear, deep, roomy space, free of the damaged edges of the membrane. (*c*) The membrane may be very little destroyed, but is adherent to the promontory and inner wall of the tympanum. (*d*) Large granulation areas or polypi may obstruct the exit of pus from the tympanic cavity. (*e*) Marked narrowing of the meatus due to chronic eczema and inflammatory pus infections of the meatal tissues is present.

3. Membrane loss, large or small, with bad drainage and in addition some inflammatory infiltration outside the limits of the middle ear cavity.

4. Cases similar to 2 and 3 but with definite symptoms and signs of labyrinthine irritation, with or without labyrinthine fistula.

*Voice Tests for Hearing.* The author's routine plan is to set the man about three feet away from him, and ask him questions regarding his rank, age, etc., so that he is unaware of being tested.

*Tuning-fork Tests.* The tests which the author employs most, and considers of the greatest value, are Rinné's and absolute bone conduction.

If bone conduction is full, Rinné's test is of considerable value in estimating middle ear deafness, and about 8 o/o of the cases one meets with are of this type.

